# The pendulum test as a tool to evaluate passive knee stiffness and viscosity of patients with rheumatoid arthritis

**DOI:** 10.1186/1471-2474-7-89

**Published:** 2006-11-29

**Authors:** Maria S Valle, Antonino Casabona, Rosaria Sgarlata, Rosaria Garozzo, Maria Vinci, Matteo Cioni

**Affiliations:** 1 Department of Physiological Sciences, School of Medicine, University of Catania, Catania, Italy; 2 Department of Internal Medicine, School of Medicine, University of Catania, Catania, Italy; 3 Department of Experimental and Clinical Pharmacology, School of Medicine, University of Catania, Catania, Italy

## Abstract

**Background:**

The pendulum test of Wartenberg is a technique commonly used to measure passive knee motion with the aim to assess spasticity. We used this test to evaluate changes of the knee angular displacement, passive stiffness and viscosity in rheumatoid arthritis patients. Stiffness and viscosity represent passive resistances to joint motion associated with the structural properties of the joint tissue and of the muscular-tendon complex. Stiffness can be considered an intrinsic property of the tissues to resist deformation, while viscosity is related to cohesive forces between adjacent layers of tissues. Both parameters may influence the joint range of motion affecting angular displacement.

**Methods:**

Nine women with rheumatoid arthritis were compared with a group of healthy women. With the subject half-lying, the relaxed knee was dropped from near-full extension and the characteristics of the ensuring damped unsustained knee oscillation evaluated. The kinematics of leg oscillations was recorded using ultrasonic markers (Zebris CMS HS 10) and the kinetic data were calculated from kinematic and anthropometric measures.

**Results:**

Knee stiffness significantly increased (p < 0.001) in patients with respect to the control group, while differences in viscosity were not significant. Moreover, the amplitudes of first knee flexion (the maximal flexion excursion after knee release) and first knee extension (the maximal extension excursion after the first knee flexion) were significantly decreased (p < 0.001). A regression analysis showed that disease severity correlated moderately with stiffness (R^2 ^= 0.68) and first flexion (R^2 ^= 0.78). Using a multivariate regression, we found that increasing stiffness was the main factor for the reduction of flexion and extension motions.

**Conclusion:**

We showed that the Wartenberg test can be considered a practical tool to measure mechanical changes of knee caused by rheumatoid arthritis. This novel application of Wartenberg test could be useful to follow up the effects of pharmacological and rehabilitative interventions in this disease.

## Background

Rheumatoid arthritis (RA) is a chronic inflammatory disease, with a prevalence of about 1% (0.2–2.0 %) on the general population. It occurs in both childhood and adulthood and women are more frequently affected than men (4/1). The main symptoms of this disease are pain and increased stiffness of the joints. RA is associated with severe morbidity, functional decline and decreased longevity [1]. The joint involvement is usually polyarticular and symmetrical and the knee is one of the joints most frequently and precociously affected. The knee joint inflammation is accompanied by progressive joint effusion, space narrowing, capsular distension, synovial hypertrophy, capsular thickening, effusion and destructive lesions of cartilage and bone, resulting in permanent joint damage. There is also evidence for pathological modifications of the muscle connective tissue. These include abnormalities in muscle fiber size and length, in muscle architecture (i.e. the angle and the physical properties of the fiber tendon attachment) and in the muscle fiber length ratio, fiber type and number of cross-bridges [2]. The clinical course of this disease is monitored by measuring some outcomes which can be more or less sensible to the progressive worsening of the motor function. The most sensible outcomes are considered the physical assessment, the grip test and the subjective measure of pain by means of an analogic scale [3]. Instead, the clinical measurement has previously been considered to have lower sensitivity because of the difficulty in its quantification.

**Figure 1:**
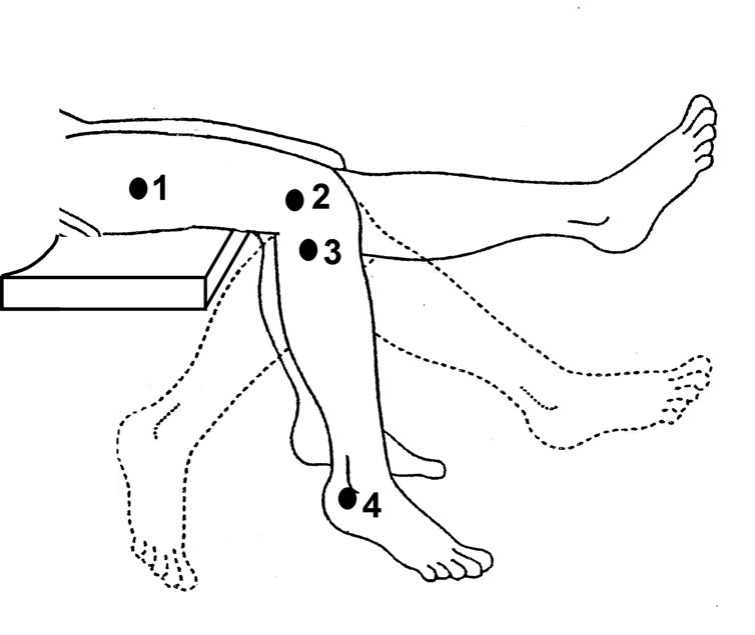
Limb oscillation during pendulum test. Solid line represents the leg at starting position (extended leg) and at final position (flexed leg). The numbers indicate location of skin reference markers: 1, 2/3 thigh; 2, lateral femoral condyle; 3, head of fibula; 4, lateral malleolus.

In spite of clinical importance of joint stiffness and of the severe anatomical modifications underlying this symptom [2], little information is available in literature about the objective evaluation of joint flexibility in RA patients (i.e. the variation of the range of joint motion). In this study we approach this issue by means of the pendulum test of Wartenberg used predominantly to measure rigidity and spasticity in neurological patients [4]. To perform the test, the clinician extends the knee and releases the limb, allowing the leg to swing passively (Fig. 1). The trajectory of the oscillating leg provides a set of kinematic parameters such as peak angular values, useful to monitor the changes in the range of knee motion.

The kinematic outcome depends on a combination of forces acting at the joint. Among these forces, stiffness and viscosity represent the passive resistances provided by the articular and periarticular tissues to the angular motion. While stiffness can be considered a generic intrinsic property of the tissue to resist deformation, viscosity is related to the friction (i.e. cohesive forces) between adjacent layers of tissues. Thus, both parameters may influence the range of motion of knee joint affecting angular displacement.

In the present work, the knee stiffness and viscosity were computed from anthropometric and angular measurements modelled following the laws of physics for a simple pendulum. Joint stiffness was then quantified in terms of the torque required to produce one unit of joint angular deflection while the viscosity was the torque required to produce one unit rate of joint angular deflection (see "Mechanical measurements and estimations" in "Methods").

With this study, we are essentially pursuing the following goals: first, we want evaluate changes in angular displacement, stiffness and viscosity in RA patients relative to healthy controls, and secondly, we seek relationships between these changes and the clinical status.

A quantitative analysis based on these parameters could be of interest to objectively measure and follow-up the progressive loss of function or the positive effects of physical or pharmacological therapy in RA patients.

## Methods

### Subjects

We tested nine women with RA (age: 52 ± 10 yrs; height: 162 ± 3.5 cm; weight: 61.5 ± 9 kg) and nine healthy women (age: 49 ± 10.5 yrs; height 162.5 ± 3.2 cm; weight 59.5 ± 5.5 kg), as control group. Four other subjects (two for each group) participated in the study, but since they were not able to relax, they were not included in the analysis. All women referred sedentary life styles and were not involved in any program of regular physical activity. The diagnosis of RA was performed at the Department of Internal Medicine, Medical School, University of Catania according to the criteria of the American Rheumatism Association (ARA) [5]. The ARA standards included the presence of morning stiffness, arthritis of three or more joints areas, arthritis of hand areas, symmetrical distribution of arthritis, rheumatoid nodules, serum rheumatoid factor and radiographic changes. Only patients with a diagnosis of RA performed by using ARA standards were included in the test group. The criteria of inclusion, concerned also the presence of a main localization at the knee joint and an age superior to eighteen years. The criteria of exclusion were the following: presence of acute exacerbation phase of RA, large popliteal cysts, previous knee surgery or traumatic orthopedic lesions of lower limbs, neurological diseases which alter the muscle tone as Parkinson's disease, stroke or cerebral palsy, and conditions of depression or anxiety (which could affect the relaxation status).

Patients were classified according to the revised criteria for functional status in RA of the American College of Rheumatology (ACR) (Table 1) [6]. This classification considers the level of functioning for the usual self-care activities (dressing, feeding, bathing, grooming and toileting), avocational (i.e. recreational or leisure) and vocational (i.e. work, school, and homemaking). These activities are patient-desired, age and gender-specific [6]. On these bases seven patients were assigned to class I and two to class III. At the moment of testing, patients were under pharmacological treatment (methotraxate, non-steroid anti-inflammatory drugs, salazopirine) and all of them had previously received corticosteroids to treat the exacerbation phases of the disease. Both clinical and/or radiological examinations confirmed bilateral involvement of the knee joint. All patients were able to walk unassisted. Before testing, all subjects participating to this study were evaluated by means of standardized clinical protocol for the range of motion of lower limbs (hip, knee and ankle), muscles strength and reflexes. At the moment of clinical examination and testing patients referred pain (mild or moderate) of knee during walking or active contraction and a mild swelling of knee was observed in most of them. To avoid morning stiffness, all patients were tested between 10.00 and 12.00 a.m. The Helsinki declaration was followed and the research protocol was approved by the Ethical Committee of the Hospital. An informed consent was obtained from all participants.

### Testing protocol

According to a testing protocol reported previously [7], the pendulum test was performed in half-lying position with the trunk inclined approximately 40° from the horizontal to provide a comfortable starting position. After lifting the relaxed lower limb to the horizontal position, the examiner released the limb and let it fall and oscillate freely between flexion and extension until it stopped (Fig. 1). The inherent viscoelastic properties of the joint and surrounding tissues, coupled with the mass of the moving foot and leg, caused the leg to finally come to rest close to the vertical position. We adapted the protocol to patients with RA and in order to avoid painful mechanical stress of the back side of the knee joint and/or reflex responses of quadriceps or hamstring muscles. The angle at the start of the test (onset angle) was chosen at a comfortable position with the knee not fully extended. It was not possible to fully extend the knee also in the control subjects because of their shortened hamstrings (evaluated during the clinical examination). This was, probably due to the age and/or the sedentary style of life of these subjects. As a consequence the onset angles of patients and control subjects were comparable over all the testing sessions (see Table 2). The same examiner tested all participants.

### Recording system

Leg movements during the pendulum test were recorded by using an ultrasonic device that continuously captured the three-dimensional spatial positions of small markers attached to anatomical reference points (CMS HS10 system Zebris, Germany). Four circular markers (7 × 6 mm DxH, 1 g) were attached on the skin over 2/3 thigh, lateral femoral condyle, head of fibula and lateral malleolus by means of double-sided adhesive patches (Fig. 1). The spatial coordinates of the markers were sampled at 100 Hz and with a spatial resolution of 0.2 mm. The data were processed by the software WinData 2.19.14, Zebris. The knee joint flexion-extension angles throughout the pendular movement were calculated from the reference marker coordinate data. Kinematic data were low-pass filtered with a zero-lag second-order Butterworth filter with 5 Hz cutoff frequency. The activity of m. rectus femoris was recorded by means of surface electromyography to ensure that participants were relaxed and that they did not try to activate this muscle to avoid some pain during knee flexion. The electromyographic activity was monitored on-line and trials showing some activity of m. rectus femoris over the baseline were rejected. Both lower limbs were examined and tests were repeated so that five successful trials were obtained for each limb.

### Mechanical measurements and estimations

**Figure 2:**
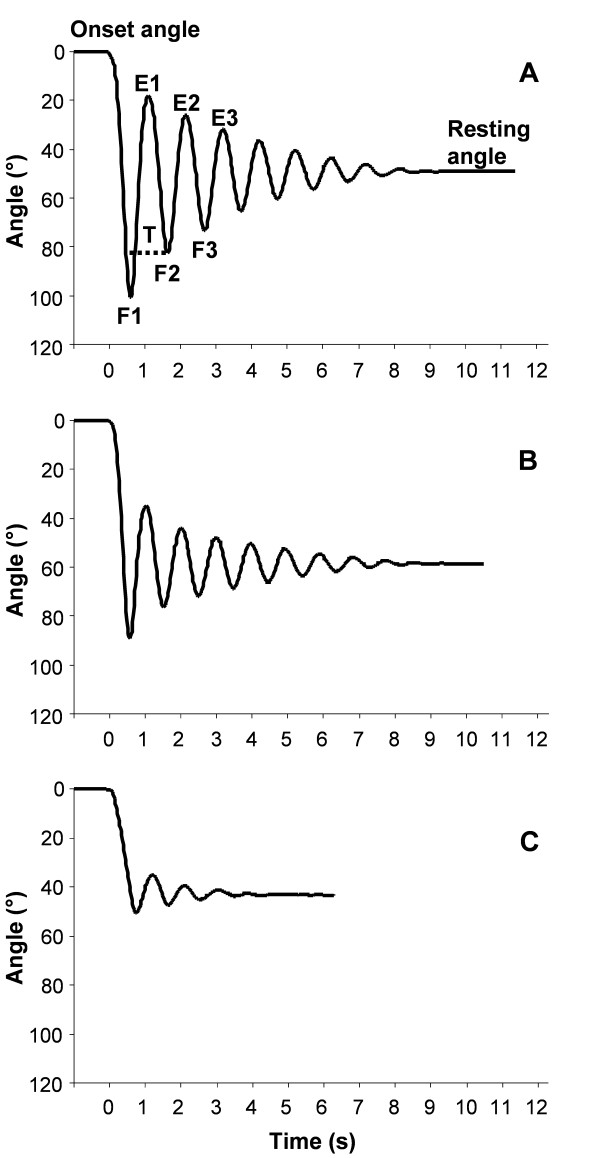
A. Typical normal knee flexion-extension angular response showing onset angle, resting angle, the first three peak flexion angles (F1, F2, F3), the first three peak extension angles (E1, E2, E3), the period of the first cycle (T); B. pendulum test angular response for a patient mildly affected (class I, ACR classification); C: pendulum test angular response for a patient severely affected (class III, ACR classification). Abbr.: ACR (American College of Rheumatology)

As shown in Fig. 2A, several variables could be derived from the kinematics of each trial of pendulum test. We measured the following displacement and timing parameters: the angle at the start of the test response (onset angle); the angle at the end of the test response (resting angle); first three peak flexion angles (F1, F2, F3); first three peak extension angles (E1, E2, E3); amplitude of initial flexion (F1Amp = F1 - onset angle); amplitude of initial extension, (E1Amp = F1 - E1); plateau amplitude (PA = resting angle - onset angle); relaxation index (RI = F1Amp/PA); extension relaxation index (ERI = E1Amp/PA); time duration from onset angle to resting angle (D); period of the first cycle (T).

Some kinematic parameters of knee motion and anthropometric measures were used to compute the variations of viscosity and stiffness during the first three oscillations.

Knee stiffness (K) and viscosity (B) were estimated by computing the damping ratio (*ζ*) and the natural frequency (*ω*) obtained from the data of each trial. We used the following equations as reported by Lin and Rymer [8]:

ζ=B2JK′=(ln⁡D)24π2+(ln⁡D)2     (1)
 MathType@MTEF@5@5@+=feaafiart1ev1aaatCvAUfKttLearuWrP9MDH5MBPbIqV92AaeXatLxBI9gBaebbnrfifHhDYfgasaacH8akY=wiFfYdH8Gipec8Eeeu0xXdbba9frFj0=OqFfea0dXdd9vqai=hGuQ8kuc9pgc9s8qqaq=dirpe0xb9q8qiLsFr0=vr0=vr0dc8meaabaqaciaacaGaaeqabaqabeGadaaakeaaiiGacqWF2oGEcqGH9aqpdaWcaaqaaiabdkeacbqaaiabikdaYmaakaaabaGaemOsaOKafm4saSKbauaaaSqabaaaaOGaeyypa0ZaaOaaaeaadaWcaaqaaiabcIcaOiGbcYgaSjabc6gaUjabdseaejabcMcaPmaaCaaaleqabaGaeGOmaidaaaGcbaGaeGinaqJae8hWda3aaWbaaSqabeaacqaIYaGmaaGccqGHRaWkcqGGOaakcyGGSbaBcqGGUbGBcqWGebarcqGGPaqkdaahaaWcbeqaaiabikdaYaaaaaaabeaakiaaxMaacaWLjaWaaeWaaeaacqaIXaqmaiaawIcacaGLPaaaaaa@4AF2@

where J is the sagittal moment of inertia applying to the leg-foot complex rotation around the knee axis; K′=K+mgl2
 MathType@MTEF@5@5@+=feaafiart1ev1aaatCvAUfKttLearuWrP9MDH5MBPbIqV92AaeXatLxBI9gBaebbnrfifHhDYfgasaacH8akY=wiFfYdH8Gipec8Eeeu0xXdbba9frFj0=OqFfea0dXdd9vqai=hGuQ8kuc9pgc9s8qqaq=dirpe0xb9q8qiLsFr0=vr0=vr0dc8meaabaqaciaacaGaaeqabaqabeGadaaakeaacuWGlbWsgaqbaiabg2da9iabdUealjabgUcaRmaalaaabaGaemyBa0Maem4zaCMaemiBaWgabaGaeGOmaidaaaaa@35FB@ and m is the leg-foot complex mass, g is the gravity acceleration and l is the distance of the leg-foot centre of mass from the knee axis; D=θ1θ2
 MathType@MTEF@5@5@+=feaafiart1ev1aaatCvAUfKttLearuWrP9MDH5MBPbIqV92AaeXatLxBI9gBaebbnrfifHhDYfgasaacH8akY=wiFfYdH8Gipec8Eeeu0xXdbba9frFj0=OqFfea0dXdd9vqai=hGuQ8kuc9pgc9s8qqaq=dirpe0xb9q8qiLsFr0=vr0=vr0dc8meaabaqaciaacaGaaeqabaqabeGadaaakeaacqWGebarcqGH9aqpdaWcaaqaaGGaciab=H7aXnaaBaaaleaacqaIXaqmaeqaaaGcbaGae8hUde3aaSbaaSqaaiabikdaYaqabaaaaaaa@3485@ that is the ratio of the peak angle of one cycle (*θ*_1_) to the peak angle of the following cycle (*θ*_2_).

ω=K′J=2πT     (2)
 MathType@MTEF@5@5@+=feaafiart1ev1aaatCvAUfKttLearuWrP9MDH5MBPbIqV92AaeXatLxBI9gBaebbnrfifHhDYfgasaacH8akY=wiFfYdH8Gipec8Eeeu0xXdbba9frFj0=OqFfea0dXdd9vqai=hGuQ8kuc9pgc9s8qqaq=dirpe0xb9q8qiLsFr0=vr0=vr0dc8meaabaqaciaacaGaaeqabaqabeGadaaakeaaiiGacqWFjpWDcqGH9aqpdaGcaaqaamaalaaabaGafm4saSKbauaaaeaacqWGkbGsaaaaleqaaOGaeyypa0ZaaSaaaeaacqaIYaGmcqWFapaCaeaacqWGubavaaGaaCzcaiaaxMaadaqadaqaaiabikdaYaGaayjkaiaawMcaaaaa@3AB3@

where T is the period of one cycle (see Fig. 2A).

The estimation for J and mass characteristics (m and l) were obtained for each subject according to Winter [9].

Using the equation (1) and (2) the values of viscosity and stiffness were obtained as follows:

*B *= 2 · *ζ *· *ω *· *J *    (3)

K=K′−mgl2     (4)
 MathType@MTEF@5@5@+=feaafiart1ev1aaatCvAUfKttLearuWrP9MDH5MBPbIqV92AaeXatLxBI9gBaebbnrfifHhDYfgasaacH8akY=wiFfYdH8Gipec8Eeeu0xXdbba9frFj0=OqFfea0dXdd9vqai=hGuQ8kuc9pgc9s8qqaq=dirpe0xb9q8qiLsFr0=vr0=vr0dc8meaabaqaciaacaGaaeqabaqabeGadaaakeaacqWGlbWscqGH9aqpcuWGlbWsgaqbaiabgkHiTmaalaaabaGaemyBa0Maem4zaCMaemiBaWgabaGaeGOmaidaaiaaxMaacaWLjaWaaeWaaeaacqaI0aanaiaawIcacaGLPaaaaaa@39C9@

On the basis of results reported by Lin and Rymer [8] and Bianchi et al. [10], the values of K and B may depend on amplitude and direction of motion that change for each half-cycle. In order to evaluate differences across the cycles of each trial, we estimated K and B for each half-cycle for the first three cycles. A half-cycle is defined by a flexion to extension (for example F1 to E1 in Fig. 2A) or an extension to flexion movement (for example E1 to F2 in Fig. 2A). Therefore, the damping ratio (equation 1) and natural frequency (equation 2) were computed from the period (T) and amplitude ratio (D) found in the experimental data for each half-cycle interval. Equation 1 can be modified for each half-cycle amplitude ratio (D) by replacing 4*π*^2 ^with *π*^2^.

The data were normalized by dividing the individual results of moment of inertia, stiffness and viscosity by the fifth power of body stature, according to a previously described procedure [11,12].

### Statistical analyses

For all subjects, means and standard errors of each parameter were calculated pooling together all trials performed on each side.

Stiffness and viscosity across the half-cycles, were modelled by a general linear model with repeated measures, using the repetition of consecutive half-cycles as within-subject factor and the condition (normal subjects vs patients with RA), as between-subject factors. Post hoc t- tests were computed for differences between the groups for each half-cycle interval. The critical value of F was adjusted by Greenhouse-Geisser procedure after checking the data for sphericity, that is, the correlations among all combinations of trials were equal. This extra test addresses a specific assumption for validate the repeated-measures ANOVA [13]. The comparison between group or side of each displacement parameters was performed by using Student's t-test.

A univariate linear regression model was performed to correlate each parameter to the grade of pathology. The following multivariate regression model was used to evaluate the dependences of amplitudes angular changes (F1amp or E1amp) to viscoelastic parameters (B or K):

F1amp [E1amp] = *β*_0 _+ *β*_1_*KF1 [KE1] + *β*_2_*BF1 [BE1] + *ε *    (5)

where KF1, KE1, BF1, BE1 represent stiffness and viscosity during the first flexion (F1) or extension (E1) oscillation, *β*_0–2 _are the regression coefficients and *ε *the residual error. The standardized regression coefficients were used to assess the contribution of each single term to the dependent variable variance. For each model, we also examined the relation between the residual errors and the predicted values for any sign of systematic trends in the residual variance.

The level of significance for all tests was set to p < 0.05. Statistical analyses were carried out using the software package SYSTAT, version 11 (Systat Inc., Evanston, IL, USA).

## Results

Leg motions of one control subject and two patients with RA are illustrated in Fig. 2. Angular values reported in the figure were normalized to the onset angle which was set to zero to better appreciate the differences of angular excursion among the displayed subjects. However, table 2 shows that the real values of onset angles measured in patients and in control subjects were similar (p > 0.05).

The amplitude of pendulum oscillations decreased and became more prolonged in patients (Fig. 2B e 2C) with respect to control subject (Fig. 2A). Changes between the two patients with RA seem to be related to ACR classification, corresponding to class I for the patient showed in Fig. 2B and class III for another patient, (Fig. 2C) who was the most affected one. The different kinematic profiles shown in Fig. 2 suggest that both viscoelastic and displacement parameters may change extensively in patients with RA. We used the data acquired from the pendulum test to quantify the effects of condition (RA vs normal) and half-cycles (first six half-cycles oscillations) on viscosity and stiffness. Differences of displacement variables between the two groups were estimated by using Student's t-test. Since in a preliminary analysis the effect of side was not found significant, to simplify the computation, we performed all the analyses on the data recorded from the right leg only.

### Stiffness and viscosity

**Figure 3:**
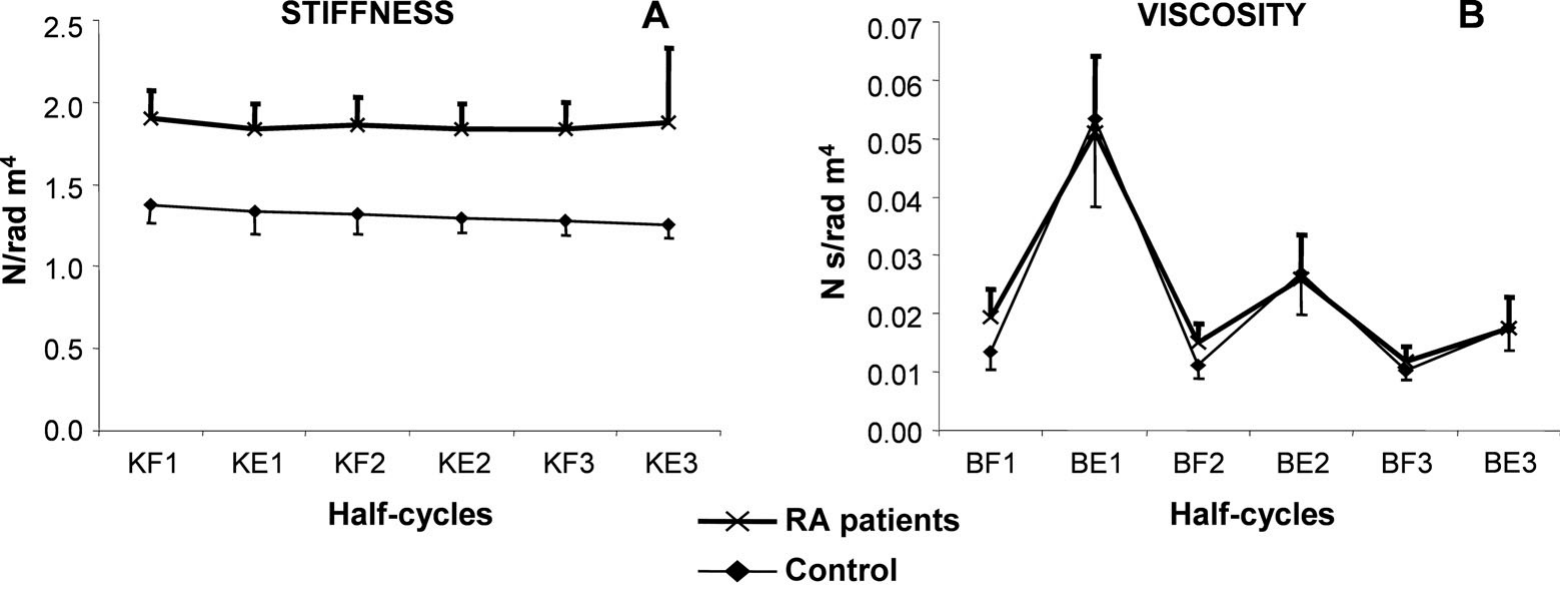
Mean values and standard errors of stiffness (A) and viscosity (B) computed for RA patients (thick line) and control group (fine line) during the first six half-cycles. KF = stiffness in flexion; KE = stiffness in extension; BF = viscosity in flexion; BE = viscosity in extension.

Each group exhibited constant values of stiffness across the half-cycles (Fig. 3A). Control group normalized stiffness ranged from 1.26 N/rad m^4 ^to 1.34 N/rad m^4^. In the patients group, the normalized stiffness changed from 1.84 N/rad m^4 ^to 1.91 N/rad m^4^. The pathological condition produced relevant effect on stiffness, which significantly increased in RA patients relative to normal subjects (about +42% over the half-cycles; F_(1,5) _= 23.66, p < 0.0001).

However, the changes of stiffness over the six half-cycles were small and were not statistically significant (F_(1,5) _= 0.73, p = 0.60). Univariate F test analysis showed that differences between the two groups, at each half-cycle, were all highly significant (p < 0.0001 for the first 5 half-cycles; p < 0.01 for the last half-cycle).

As it can be seen in the Fig. 3B, the viscosity values fluctuated and progressively decreased from the beginning to the end of leg oscillations. All over the cycles, there was a directional bias since viscosity values were greater in flexion than in extension. The normalized values of viscosity changed in normal subjects from 0.010 N sec/rad m^4 ^to 0.053 N sec/rad m^4^, whereas in patients with RA ranged from 0.012 N sec/rad m^4 ^to 0.051 N sec/rad m^4^. We did not observe any significant differences between the patients with RA and the control group for the pathological condition (F_(1,5) _= 0.013, p = 0.910). Instead, viscosity displayed large changes over the six half-cycles (F_(1,5) _= 70.06, p < 0.0001) according to flexion-extension movement direction.

In the table 2 are reported, for each participant, the values of stiffness (KF1) and viscosity (BF1) concerning the first half-cycle (the first flexion).

### Displacement parameters

**Figure 4:**
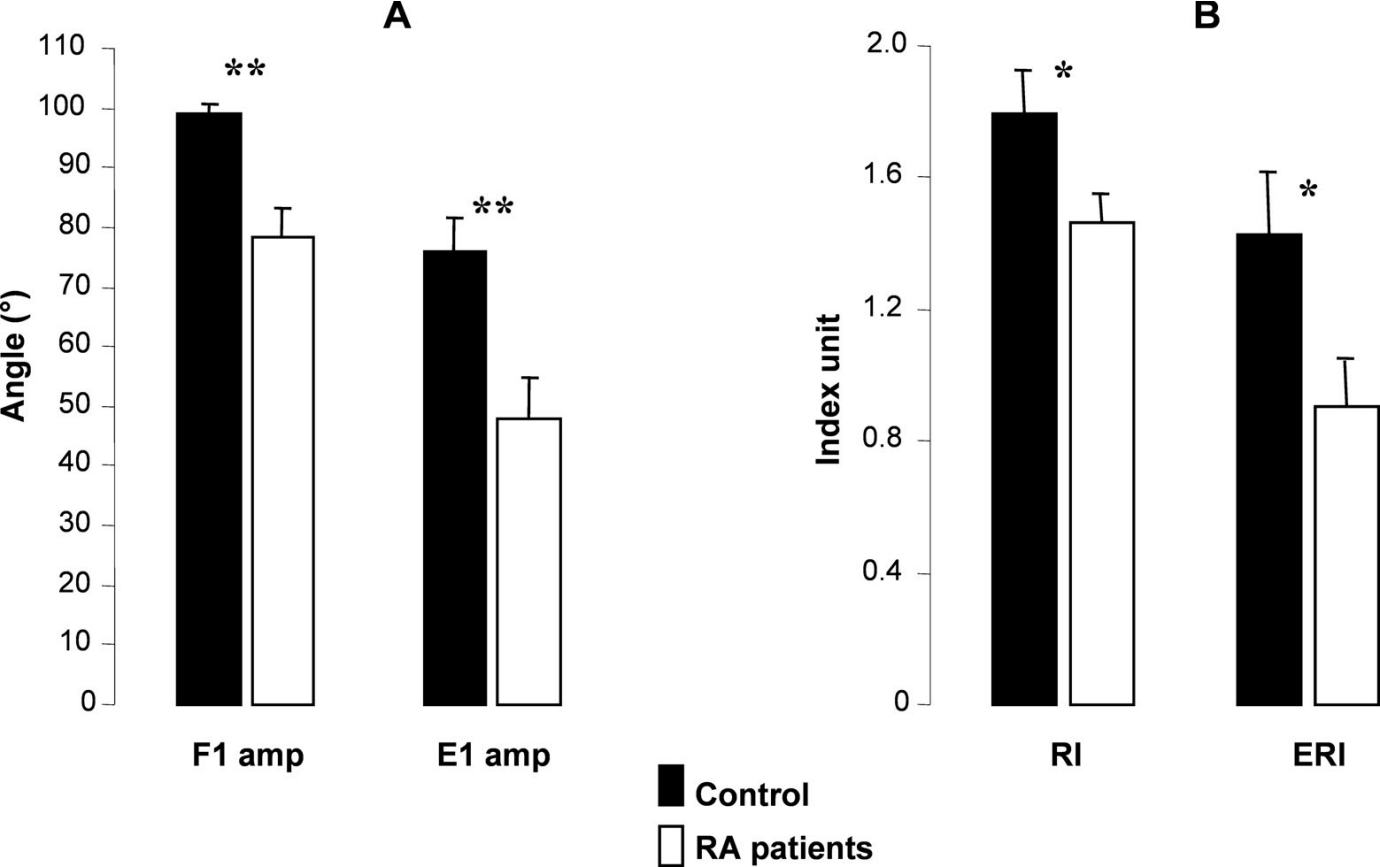
Mean values and standard errors of F1amp (amplitude of first flexion), E1amp (amplitude of first extension), RI (relaxation index) and ERI (extension relaxation index). * = p < 0.05, ** = p < 0.01.

Among all displacement parameters measured, only F1Amp, E1Amp, RI and ERI exhibited statistically significant changes between normal subjects and patients (Fig. 4 and Table 2). Relative to the control subjects, patients with RA showed significant reductions of the mean values of F1Amp (-21%, p < 0.001, Fig. 4A), E1Amp (-37%, p < 0.001, Fig. 4A), RI (18%, p < 0.05, Fig. 4B) and ERI (-37%, p < 0.05; Fig. 4B). Mean values of onset angle, resting angle, plateau amplitude and duration were not significantly different between the two groups (p > 0.05). In the latter case the result was influenced by the high variability of duration of oscillations detected in the patient group. Because some of the displacement or temporal parameters could be correlated to each other, results may in part reflect correlations across measured parameters. For example, absence of significant variations of onset and resting angle restricted the variations of PA (PA = resting angle - onset angle). Therefore, comparable values of PA in the two groups linked the resulting values of RI and ERI to the measures of F1Amp and E1Amp, respectively (RI and ERI indexes were computed from the ratio of F1Amp and E1Amp to the PA; see Methods).

### Results of regression analysis

**Figure 5:**
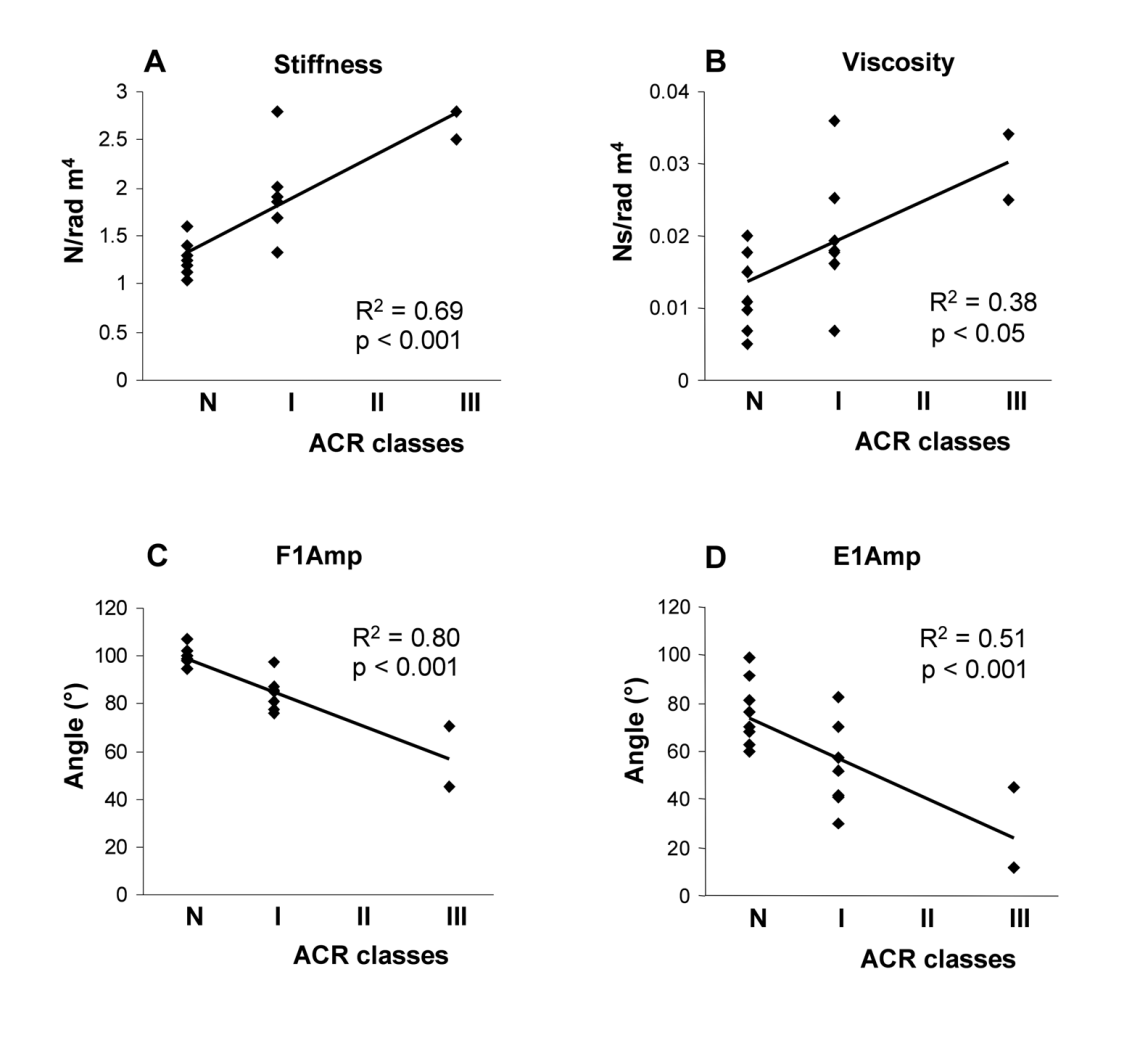
Results of univariate linear analyses. The normal condition (N) and the pathology severity (ACR classification, [6]) were plotted against stiffness (A), viscosity (B), F1amp (C) and E1amp (D). R^2 ^= coefficient of determination; N = normal subjects.

The influence of severity of RA on the single displacement or viscoelastic parameters was evaluated by using a univariate linear regression analysis. The results are illustrated in Fig. 5 where the normal condition (N) and the pathology severity, quantified with the ACR classification, were plotted against each single parameter (data points represent average values across trials for each subject). The highest coefficients of determination were showed by F1Amp (R^2 ^= 0.80, Fig. 5C), stiffness (R^2 ^= 0.69, Fig. 5A) and E1Amp (R^2 ^= 0.51, Fig. 5D) measured in the first half-cycle, whereas the other parameters were weaker predictors of RA severity (viscosity: R^2 ^= 0.38, Fig. 5B; RI: R^2 ^= 0.36; ERI: R^2 ^= 0.33).

**Figure 6:**
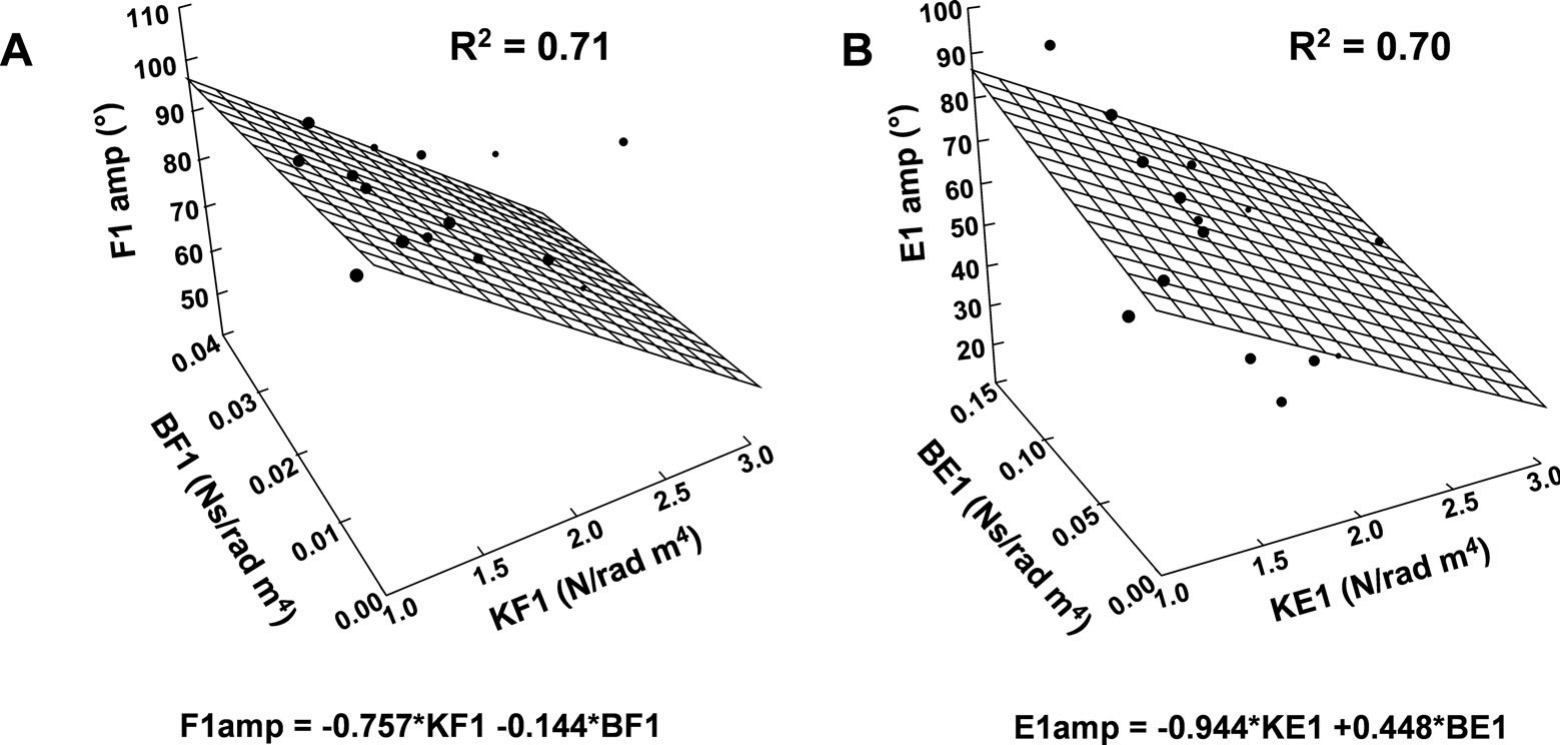
Multivariate linear analysis to evaluate (A) the relationship between F1amp (amplitude of first flexion) and BF1 and KF1 (viscosity and stiffness during first flexion, respectively); (B) the relationship between E1amp (amplitude of first extension) and BE1 and KE1 (viscosity and stiffness during first extension, respectively). Regression model, represented by the surface grid, is superimposed on the observed data.

The reduction in knee angle excursion observed in patients with RA during the first cycle depended on the combined increasing of stiffness and viscosity. We evaluated the contribution of these two kinetic parameters on range of motion decrease by means of a multivariate linear regression (see equation 1 in Methods). The results of this analysis are displayed in Fig. 6. In this figure, the regression fit to the data is represented by the surface grid, which shows that the fits were comparable for both displacement parameters (F1amp: R^2 ^= 0.71, Fig. 6A; E1amp: R^2 ^= 0.70, Fig. 6B). The plots of the regression fits and of the associated regression coefficients indicate that during the first two half-cycles (KF1 and KE1), stiffness was the best predictor for both F1Amp and E1Amp variations. In fact, the rate of changes along the stiffness axis was greater than the rate along the viscosity axis, that is the standardized regression coefficient was higher for stiffness than for viscosity (-0.757 vs -0.144 for F1amp, Fig. 6A; -0.944 vs 0.448 for E1 amp, Fig. 6B).

## Discussion

In this study we showed that the pendulum test of Wartenberg can be used to estimate changes of passive knee stiffness and viscosity in patients with RA. The analysis of limb oscillations during this test showed that the amplitudes of first flexion and first extension movements were markedly reduced in these patients, coinciding with a significantly increased knee stiffness. The correlations found between the clinical status and the biomechanical parameters (in particular stiffness, F1 Amp and E1 Amp) suggest that these parameters could be reliable markers of disease severity, potentially useful to follow-up the evolution of this chronic disease.

Wartenberg [4] introduced the pendulum test as a simple and effective method of evaluating the tone of knee extensor muscles (especially spasticity) in patients with neurological diseases. It can be performed by a simple tool as an electrogoniometer [14,15] or more complex video-based systems [7] and it was validated in healthy adults [16]. Some quite recent studies have shown that the pendulum test outcomes are well correlated with clinical findings [17] and sufficiently precise to evaluate lower limbs hypertonia of individuals with cerebral palsy [18] as well as of those with Parkinson's disease [16]. Furthermore, this test has been recognized as an accurate tool to evaluate the pharmacological treatment of spasticity [10] or the effects of surgical intervention of selective dorsal rhizotomy in children with spastic diplegia [19].

Whilst the amplitudes of flexion and extension joint excursions are common parameters measured in most of the studies based on the Wartenberg test, a combination of the kinematic data with anthropometric data allows for the derivation of useful kinetic data. In fact, passive leg oscillations may be modelled according to the laws of physics for a simple pendulum and the computation of natural frequency (*ω*) and damping ratio (*ζ*) can support evaluation of the stiffness and viscosity associated with the cyclic movement [8-10] (see "Mechanical measurements and estimations" in "Methods").

In this study, the main contribution to the range of motion reduction in the patients came from modifications in stiffness which appears to be a good predictor of the disease severity.

Joint stiffness can be expressed in terms of torque required to produce one unit of passive angular deflection and might result from the sum of the resistances of many tissues such as ligaments, tendons, cartilages, muscles and bones [20-22]. The contribution of articular and periarticular tissues to passive joint stiffness varies with the amplitude of motion [23]. Although the present results cannot provide valuable information about this issue, some indication are given by Johns and Wright that investigated small healthy human joints (eg. metacarpophalangeal) [23]. These authors showed that the tendons stiffness increased mainly at the extremes of joint motion while joint capsule and muscles accounted for most of the resistance produced in the midrange of joint motion.

Measured stiffness values can be influenced by several factors, mainly the changes in motor task and the modalities of measurement. For example, reflex or voluntary muscle contractions are possible sources of ambiguity since they make it difficult to distinguish between the intrinsic stiffness produced by passive components and the stiffness transiently modulated by neural control [22]. In addition, the specificity of the RA pathophysiology may increase the inaccuracy in measuring stiffness values. In fact, if the biomechanical deformities cause an objective quantifiable increase of stiffness, the painful experience and a possible impaired perception of the proprioceptive signals [24-28] might induce a misrepresentation of the sense of joint motion affecting the movements.

Therefore, it is important to check the subject behaviour over the period of the test and to standardize the procedures. In this study, the continuous EMG monitoring to prevent muscle activation and the typical non-weightbearing conditions in which the test was performed, greatly minimized the disturbances created by active components or due to the discomfort for the painful experience. Thus, the clear differences in stiffness values between healthy subjects and RA patients reported in this study depend mainly on the authentic passive knee stiffness and the Wartenberg test could be considered a practical method to obtain objective measures of this parameter.

With respect to the subjective sense of stiffness discussed by several authors [24-28], the present results cannot provide useful insights into the nature of this sensation in RA. However, the possibility to use the Wartenberg test for a quantitative estimation of passive joint stiffness might form an interesting basis for further study on the relationships between the objective and the perceived magnitude of stiffness.

The total resistance produced by joint tissues may also depend on viscosity, that is, the frictional force between adjacent layers of tissues. We have shown small changes of viscosity in RA patients relative to healthy subjects and the correlation of this parameter with F1amp and E1amp was smaller than the correlation with the stiffness. Thus, the joint damage produced by RA disease would affect the overall ability to resist deformation of articular and periarticular tissues, with less significant contribution by the resistance yielded by cohesion between adjacent layers.

The time-varying viscosity observed across half-cycles for both normal and patients might depend on the thixotropy properties of joint tissues. Thixotropy is a phenomenon that accounts for a temporary reorganization of the internal structure following a perturbation such as the periodic knee motion in our protocol. Thixotropic behaviour has been described in skeletal muscle [29] and some authors suggested that it is a feature of the joint synovial fluid [30-32]. Thus, the periodic changes in viscosity reported here could partly rely on tissue thixotropy that was exhibited both by patients with RA and normal subjects. Very few studies compared changes in thixotropy of synovial fluid between normal and patients with RA and the results reported are contradictory. Altmann et al. [33] compared the mechanics of synovial fluid in several pathological conditions and their results indicated that thixotropy bears little diagnostic significance. Later, Safari et al. [32] (1990) demonstrated differences of synovial fluid viscosity between patients with RA and normal subjects. Since in this latter paper the measurements were performed on samples of synovial fluid aspirated from the knee joint, we cannot compare these data with those of the present study where the experimental design included complex interactions across joints. Thus, the presence of viscosity periodic changes in RA needs further investigations.

## Conclusion

The data reported in this study support the application of the Wartenberg test in the field of clinical rheumatology to identify possible changes in knee angular displacement and in knee stiffness in patients with RA. This could be useful to follow-up both the natural history of this disease and the effects of pharmacological and physical therapy interventions. This test is simple and can be performed by means of either an electrogoniometer or more complex video motion systems, both of which have become increasingly available throughout the clinical environment. Furthermore, being a test under non-weightbearing conditions, the pendulum test is likely to cause less discomfort to patients than weightbearing activities such as walking and negotiating stairs.

## Competing interests

The author(s) declare that they have no competing interests.

## Authors' contributions

MSV participated in the design of the research project, performed the recording sessions, and drafted the manuscript. AC participated in the design of the research project, performed the statistical analysis, helped to draft the manuscript. RS participated in the design of research project, helped during the recording sessions, performed the clinical evaluations and enrolled patients. RG participated in the design of the research project and performed the clinical evaluations. MV participated in the design of research project, performed the clinical evaluations. MC: conceived the study and participated in its design, coordinated the project and helped to draft the manuscript.

All authors read and approved the final manuscript.

## Pre-publication history

The pre-publication history for this paper can be accessed here:



## Figures and Tables

**Table 1:** Revised criteria for functional status in RA (American College of Rheumatology) [6].

Class	Functional status
I	Completely able to perform usual activities of daily living (self care, vocational and avocational)
II	Able to perform usual self-care and vocational activities but limited in avocational activities
III	Able to perform usual self-care activities but limited in vocational and avocational activities
IV	Limited in ability to perform usual self-care, vocational and avocational activities

Modified from Hochberg et al., 1991 [6]

**Table 2:** Summary of results concerning the onset angle, the amplitude of initial flexion (F1Amp), the amplitude of initial extension (E1Amp), the relaxation index (RI), the extension relaxation index (ERI), the stiffness (KF1) and the viscosity (BF1) during the first flexion.

control nr.	onset angle (°)	F1amp (°)	E1amp (°)	RI	ERI	KF1^a ^(N/rad m^4^)	BF1^a ^(N sec/rad m^4^)
1	9.9	94.4	76.5	2.0	1.62	1.51	0.018
2	10.3	100.3	98.9	2.4	2.33	1.13	0.021
3	15.7	106.7	91.1	2.0	1.71	1.90	0.020
4	11.7	98.6	62.8	1.4	0.90	1.31	0.007
5	10.9	98.1	60.0	1.4	0.87	1.05	0.005
6	13.0	94.6	68.3	1.6	1.12	1.40	0.010
7	10.3	99.0	69.9	1.8	1.43	1.28	0.015
8	18.5	102.0	80.9	1.8	1.41	1.23	0.011
9	11.6	100.1	99.0	2.1	1.70	1.06	0.020
							
mean	12.4	99.3	78.6	1.8	1.46	1.32	0.014
SE	1.0	1.2	5.0	0.1	0.15	0.09	0.002

patient nr.							

1	15.4	45.5	41.1	1.3	0.40	2.45	0.023
2	10.1	70.3	61.1	1.5	0.77	2.29	0.033
3	10.4	77.3	41.7	1.4	1.23	1.79	0.035
4	9.1	97.3	30.1	1.2	1.65	1.19	0.018
5	19.2	87.3	11.8	1.6	1.45	1.84	0.024
6	12.8	85.5	57.2	1.7	0.63	1.88	0.007
7	10.0	85.0	75.1	1.9	0.87	2.51	0.019
8	13.8	80.9	76.2	1.6	0.72	1.58	0.018
9	15.1	75.8	44.7	1.2	0.50	1.69	0.016
							
mean	12.9	78.3	48.8	1.5	0.91	1.91	0.021
SE	1.1	4.8	7.0	0.1	0.14	0.14	0.003

^a ^Parameters normalized by fifth power of body height
